# Situational and Dispositional Achievement Goals’ Relationships with Measures of State and Trait Sport Confidence: A Systematic Review and Meta-Analysis

**DOI:** 10.3390/ejihpe16020018

**Published:** 2026-01-30

**Authors:** Hannah Quick, Marc Lochbaum

**Affiliations:** 1 Department of Exercise and Sport Sciences, Lubbock Christian University, Lubbock, TX 79407, USA; hquick7009@lcu.edu; 2 Department of Kinesiology and Sport Management, Texas Tech University, Lubbock, TX 79409, USA; 3 Education Academy, Vytautas Magnus University, 44248 Kaunas, Lithuania

**Keywords:** motivational climate, ego and task goal orientation, self-belief, competitive sport, TEOSQ, POSQ

## Abstract

The purpose of this systematic review and meta-analysis (PROSPERO ID: CRD42024575181) was to quantify the relationships between dispositional and situational achievement goal involvement and sport confidence. A secondary purpose was to examine potential moderators of these relationships. Published studies reporting sufficient data, including one achievement goal measure from the dichotomous framework and one measure of sport confidence in an athlete sample, were included. Information sources included EBSCOhost databases, Web of Science databases, and relevant meta-analyses. The random-effects correlational coefficient (*r*) served as the summary statistic. Thirty-six studies yielding 37 independent samples, published between 1988 and 2026, which met all inclusion criteria, representing a total of 10,461 participants from youth to elite sports across four continents. Meta-analyzed random-effects correlations between task climate (k = 15, *r* = 0.33 [95% CI 0.23, 0.43]), ego climate (k = 13, *r* = −0.08 [95% CI −0.16, −0.00]), task orientation (k = 26, *r* = 0.27 [95% CI 0.21, 0.32]), ego orientation (k = 26, *r* = 0.11 [95% CI 0.06, 0.17]), and sport confidence ranged from small and negative to medium and positive in magnitude. Mixed-effects moderator analyses revealed significant differences (*p* < 0.05) for task climate when comparing state (*r* = 0.24) versus trait (*r* = 0.41) sport confidence measures, for task orientation scale (TEOSQ r = 0.31 vs. POSQ *r* = 0.18) in relation to sport confidence, and for study quality (lowest *r* = 0.35, medium *r* = 0.18, highest *r* = 0.24) in the task orientation–sport confidence relationship. However, nearly all prediction intervals for the examined relationships crossed zero, with the exception of a few TEOSQ- and POSQ-based moderator analyses. Thus, researchers and practitioners are cautioned that relationships between dispositional achievement goals, motivational climate perceptions, and sport confidence might be minimal or vary based on the dispositional achievement goal measure.

## 1. Introduction

AGT, including perceived motivational climate and dispositional goals, relates to a variety of sport-based behaviors, emotions, and cognitions (Biddle et al., 2003; Harwood et al., 2015; Lochbaum et al., 2016a, 2016b; Lochbaum & Sisneros, 2024a, 2024b). A researched concept in the sport psychology literature is sport confidence. As early as the late 1980s, research linking goal orientations and sport confidence appeared (Vealey & Campbell, 1988). Recent research suggests the importance of studying goal orientations and sport confidence together to best understand sport performance (Wu et al., 2025). Motivational climate and sport confidence appear to be studied less often than goal orientations (Harwood et al., 2015). However, there appears to be an increase in motivational climate and sport confidence research since Harwood and colleagues’ meta-analysis (Fry et al., 2021) and studies not included (Gillham et al., 2013). Thus, to date, there has been no comprehensive quantitative review of AGT and sport confidence in the sport psychology literature. Therefore, the purpose of this systematic review is to quantify the relationships between AGT measures and sport confidence in competitive athletes and explore potential moderators of these relationships.

AGT is a prominent motivational framework. Beginning in the 1980s, with Nicholls’ (1984) framework as a foundation, AGT began to play an increasingly significant role in sport psychology. The dichotomous AGT framework conceptualizes an individual’s achievement goal involvement as being influenced both by their dispositional goal orientation tendencies and by external situational factors, such as the motivational climate created by coaches or parents (Duda & Nicholls, 1992; Roberts, 1992; Selfriz et al., 1992). Task or mastery involvement and ego or performance involvement are characterized by the desire to either develop or demonstrate competence. When athletes evaluate success or failure based on self-referenced performance measures, they are demonstrating a task orientation. Similarly, if athletes perceive that their environment emphasizes personal mastery and improvement, they are operating in a mastery climate. In contrast, athletes display an ego orientation when they evaluate success or failure based on other-referenced performance measures. If the athlete’s environment also defines success as beating opponents and demonstrating competence, they are operating within an ego climate.

Sport confidence has been defined as “the belief or degree of certainty individuals possess about their ability to be successful in sport” (Vealey, 1986, p. 222). Sport confidence has been a central topic in sport psychology research, with single studies appearing in the research literature around the same time as AGT (e.g., Gayton & Nickless, 1987; Gould et al., 1981, 1984). These early studies were based on initial measures of sport confidence from Martens and his doctoral students at the University of Illinois (Martens et al., 1983) and then his former students as independent researchers (Vealey, 1986). Early work provided the field with state measures, such as the Competitive State Anxiety Inventory-2 (CSAI-2, Martens et al., 1983) and the State Sport-Confidence Inventory (SSCI, Vealey, 1986), and trait measures, such as the Trait Sport-Confidence Inventory (TSCI, Vealey, 1986) and the more recent Athlete Engagement Questionnaire (AEQ, Lonsdale et al., 2007). Regarding AGT and sport confidence, Vealey and Campbell (1988) appeared to be the first to research AGT and sport confidence, with Mills continuing this line of research (Mills, 1996a, 1996b).

### Study Purposes and Hypotheses

Though researchers have investigated AGT and sport confidence for decades, no meta-analysis has yet focused on sport confidence with only athlete samples. For instance, past AGT meta-analyses (Harwood et al., 2015; Lochbaum et al., 2016b) have grouped related variables such as confidence, perceived competence, and self-esteem. Hence, the purpose of this systematic review and meta-analysis was to quantify the relationships between situational and dispositional measures from the dichotomous AGT framework and confidence in athletes.

We formed our hypotheses based on the results from the previously discussed achievement goal meta-analyses (Harwood et al., 2015; Lochbaum et al., 2016b; Lochbaum & Sisneros, 2024a, 2024b) and confidence meta-analyses (Craft et al., 2003; Woodman & Hardy, 2003; Lochbaum et al., 2022b; Jekauc et al., 2023). Specifically, we anticipated positive correlational relationships between the task constructs and confidence (Harwood et al., 2015; Lochbaum et al., 2016a, 2016b; Lochbaum & Sisneros, 2024a), a small positive relationship between ego orientation and confidence (Lochbaum et al., 2016b), and a small negative correlational relationship between an ego climate and confidence (Harwood et al., 2015). As for potential moderator analyses, at the outset, we were unsure of whether we had enough samples to adequately test moderators, as we did not anticipate the numerous articles that we found in the Lochbaum and Sisneros (2024a) meta-analyses, with 82 articles examining motivational climate and hedonic well-being constructs. However, we chose logical moderator variables to explore, with some support in the research suggesting that state (mainly CSAI-2 confidence scale) versus trait confidence measures might be of importance (Woodman & Hardy, 2003), as well as the sex makeup of the samples (Lochbaum et al., 2022b; Woodman & Hardy, 2003), sport type (Craft et al., 2003), athlete level (Lochbaum & Sisneros, 2024a, 2024b; Woodman & Hardy, 2003), and the goal measure (e.g., TEOSQ or POSQ, Lochbaum et al., 2016a, 2016b).

## 2. Materials and Methods

This systematic review and meta-analysis followed the Preferred Reporting Items for Systematic Reviews and Meta-Analyses (PRISMA) guidelines (Page et al., 2021) and the checklist is located in Table S1. The formulation, computation, and interpretation of results were guided by Borenstein, Hedges, Higgins, and Rothstein’s Comprehensive Meta-Analysis (CMA) Version 4 program, along with its statistical output and interpretive texts (Borenstein, 2019; Borenstein et al., 2021, 2022).

### 2.1. Eligibility Criteria

The included studies met the following criteria: (a) participants were involved in competitive sports; (b) a dichotomous situational or dispositional achievement goal measure was included; (c) a sport confidence measure was included; (d) there was sufficient data to enter into the CMA program; and (e) the study was published in a peer-reviewed scholarly journal. We excluded studies that were not conducted in a competitive sports environment, were experimental (e.g., manipulated goal climate), or which did not include at least one measure of dichotomous achievement goals or confidence. Though we searched using only English terms, we did not exclude non-English articles that appeared in the search that were retrieved due to their English titles, keywords, or abstracts. For these studies, we used Google Translate to identify and translate non-English articles.

### 2.2. Information Sources, Search Strategy, and Search Protocol

**Figure 1:**
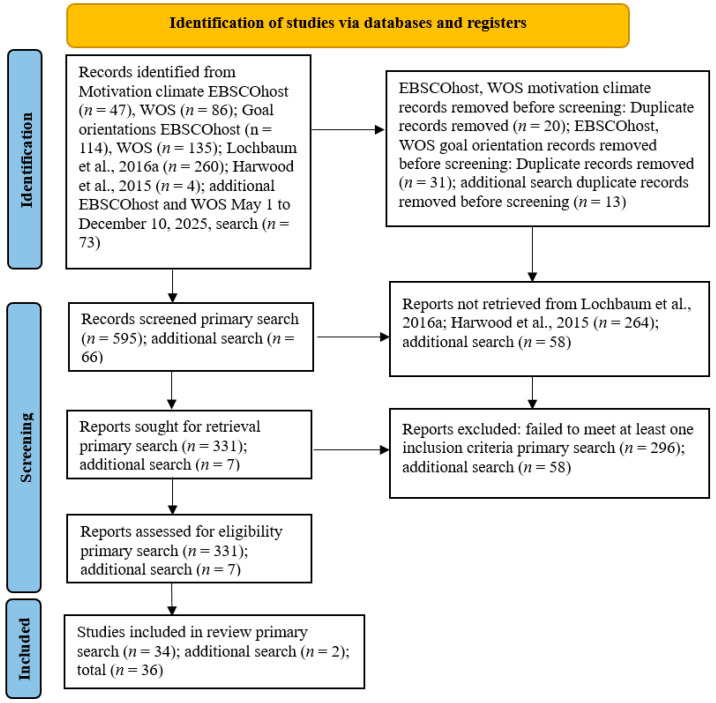
PRISMA flow chart for the identification of included articles. Figure citations: (Harwood et al., 2015; Lochbaum et al., 2016a).

Information sources consisted of databases within EBSCOhost and Web of Science, and two meta-analyses. Figure 1 details the search process and sources. The following search terms were used: goal orientation or goal orientations or achievement goals or achievement goal AND confidence AND sport*; motivational climate or motivation climate AND confidence AND sport*. The first author conducted the primary search, and the second author conducted the supplemental search (covering 1 May to 10 December 2025), given the length of time taken by the initial review and then submitted to the current journal. In the second search, the search terms were combined as (goal orientation or goal orientations or achievement goals or achievement goal or motivational climate or motivation climate) AND confidence AND sport*. Together, the authors examined the search results.

### 2.3. Data Retrieved

Jointly, both authors retrieved the following data: participant characteristics (mean age, percent female, level of participation, country), study characteristics (design, time frame of AGT and confidence measurement), AGT and confidence measure characteristics (name of measure, measure reference), data, and the study citation. To classify participant level, we used Lochbaum et al.’s (2022a) system (see Table S2) based on Kyllo and Landers (1995) and Swann and colleagues (Swann et al., 2015).

### 2.4. Study Quality Rating Scale

**Figure 2:**
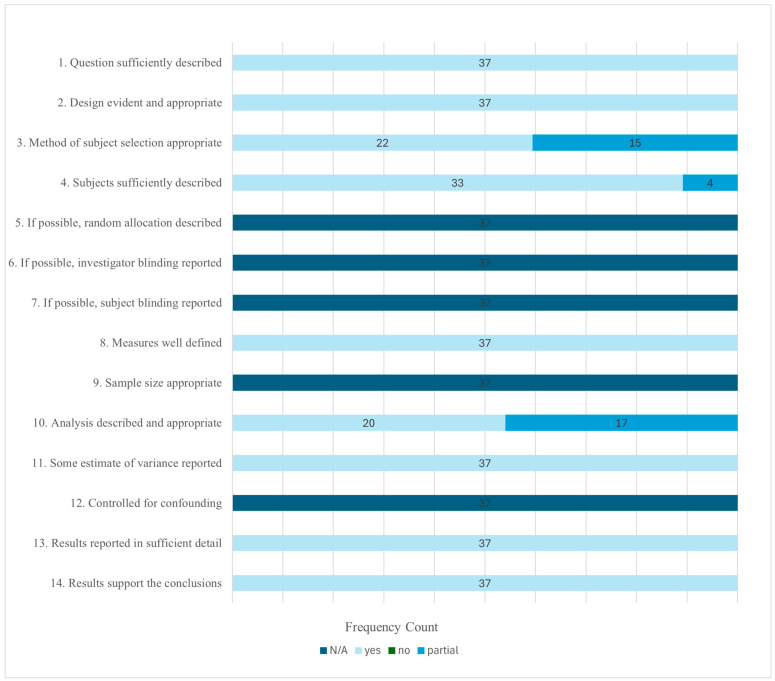
Study quality frequency counts by question.

The Kmet et al. (2004) quality system and scoring criteria were used. Each author rated sets of questions individually and consulted each other when questions arose during the rating process. Figure 2 and a table in the Supplemental file (Table S3) describe Kmet and colleagues’ questions.

### 2.5. Risk of Bias Statistics

The following risk of bias statistics were reported: the classic fail-safe *n* (Rosenthal, 1979), Orwin’s (1983) fail-safe *n*, and a funnel plot with Duval and Tweedie’s (2000) trim and fill. Table S4 contains a description of each statistic. The accuracy of these measures depends on the search for studies and the data entered.

### 2.6. Summary Statistics and Planned Analyses

The random effects correlation coefficient was used as the reported summary statistic. For interpretation, we followed Cohen’s (1990) guidelines: 0.10–0.29 as small, 0.30–0.49 as medium, and 0.50 as large for r. The following statistics were reported for our relationships: the number of samples (*k*), *r*, 95% confidence and prediction intervals, Tau-squared (τ^2^), I-squared (*I*^2^), and publication bias statistics. For each study, only one summary statistic per AGT construct (i.e., task orientation, ego orientation, task climate, and ego climate) was reported. For example, if a study reported multiple correlations for one of the relationships, such as task orientation and sport confidence, those correlations were combined into one effect size value. To test categorical moderators with sufficient samples (e.g., study quality, athlete level, confidence measure, state, or trait), a mixed-effects model analysis was used. Meta-regression was used for the sample sex makeup based on percent female. For the moderator analysis, we set the significance at the traditional *p* < 0.05. Last, the remove-one study analysis examined the robustness of our relationships beyond the two fail-safe statistics.

## 3. Results

### 3.1. Study Characteristics

Table 1 presents details of the 36 included studies, with one study providing two independent samples (k = 37). The publication years of the included studies spanned from 1988 to 2026, with the following frequencies of studies per decade: 1980s (*n* = 1), 1990s (*n* = 4), 2000s (*n* = 4), 2010s (*n* = 14), and 2020s (*n* = 13). Samples were reported from countries across four continents as follows: Europe (Austria, Germany, Hungary, Norway, Portugal, Spain, and the UK), Asia (China, Israel, Malaysia, Pakistan, and Turkey), North America (Mexico and the USA), and South America (Brazil). The studies included a total of 10,461 participants, with sample sizes ranging from 37 to 1795 participants per study. Participants competed at each level based on the Lochbaum et al. (2022a) coding system: elite (*n* = 5), advanced (*n* = 12), intermediate (*n* = 10), recreational (*n* = 1), youth (*n* = 3), and mixed (*n* = 6). Participants competed in both individual sports (e.g., fencing, swimming, and wrestling) and team sports (e.g., beach volleyball, field hockey, and soccer). Of the 36 samples, 34 reported the male and female composition with a collective mean of 44.85% female participants, with 14 samples having more than 50% female participants. Table S5 describes our assessment (i.e., state or trait) for the sport confidence measures found in each study.

### 3.2. Study Quality

Using the Kmet et al. (2004) quality assessment rating system, all samples were rated based on questions 1–4, 8, 10, 11, 13, and 14 (see Figure 2). Since no experimental studies were included, questions 5–7, 9, and 12 were scored as N/A for all samples. The quality score of the studies had a mean summary score of 0.94 (SD = 0.04). The median score was 0.94. Individual study quality scores ranged from 0.89 to 1.00, indicating that the studies were of satisfactory quality. Three distinct groups emerged: studies with two partial ratings, those with one partial rating, and those with only yes ratings. Based on the three groups, the groupings were tested as to their impact on the primary study results.

### 3.3. Individual Study Data, Synthesis of Results, and Risk of Bias Across Studies

**Figure 3:**
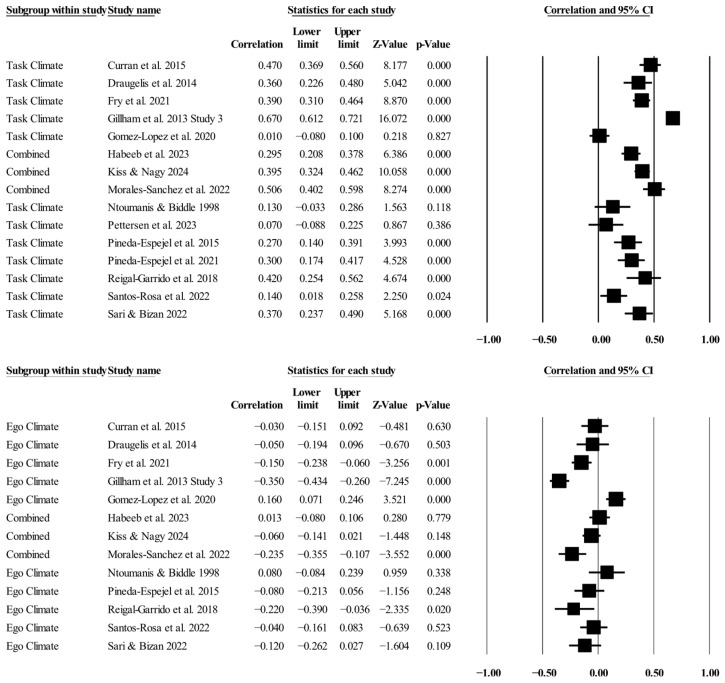
Task and ego climate and sport confidence statistics with corresponding forest plots. Combined means more than one data point entered for the listed study. Figure citations: (Curran et al., 2015; Draugelis et al., 2014; Fry et al., 2021; Gillham et al., 2013; Gómez-López et al., 2020; Habeeb et al., 2023; Kiss & Nagy, 2024; Morales-Sanchez et al., 2022; Ntoumanis & Biddle, 1998; Pettersen et al., 2023; Pineda-Espejel et al., 2015; Pineda-Espejel et al., 2021; Reigal-Garrido et al., 2018; Santos-Rosa et al., 2022; Sarı & Bizan, 2022).

**Figure 4:**
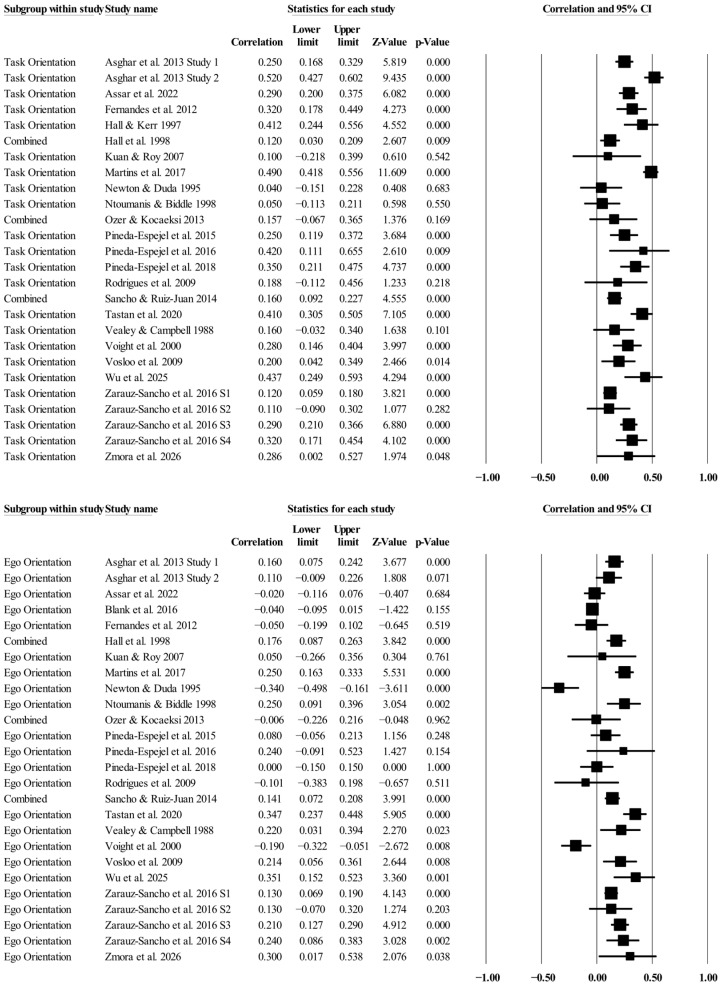
Task and ego orientation and sport confidence statistics with corresponding forest plots. Combined means more than one data point entered for the listed study. Figure citations: (Asghar et al., 2013; Assar et al., 2022; Fernandes et al., 2012; Hall & Kerr, 1997; Hall et al., 1998; Kuan & Roy, 2007; Martins et al., 2017; Newton & Duda, 1995; Ntoumanis & Biddle, 1998; Ozer & Kocaeksı, 2013; Pineda-Espejel et al., 2015; Pineda-Espejel et al., 2016; Pineda-Espejel et al., 2018; Rodrigues et al., 2009; Sancho & Ruiz-Juan, 2014; Tastan et al., 2020; Vealey & Campbell, 1988; Voight et al., 2000; Vosloo et al., 2009; Wu et al., 2025; Zarauz-Sancho et al., 2016; Zmora et al., 2026).

**Figure 5:**
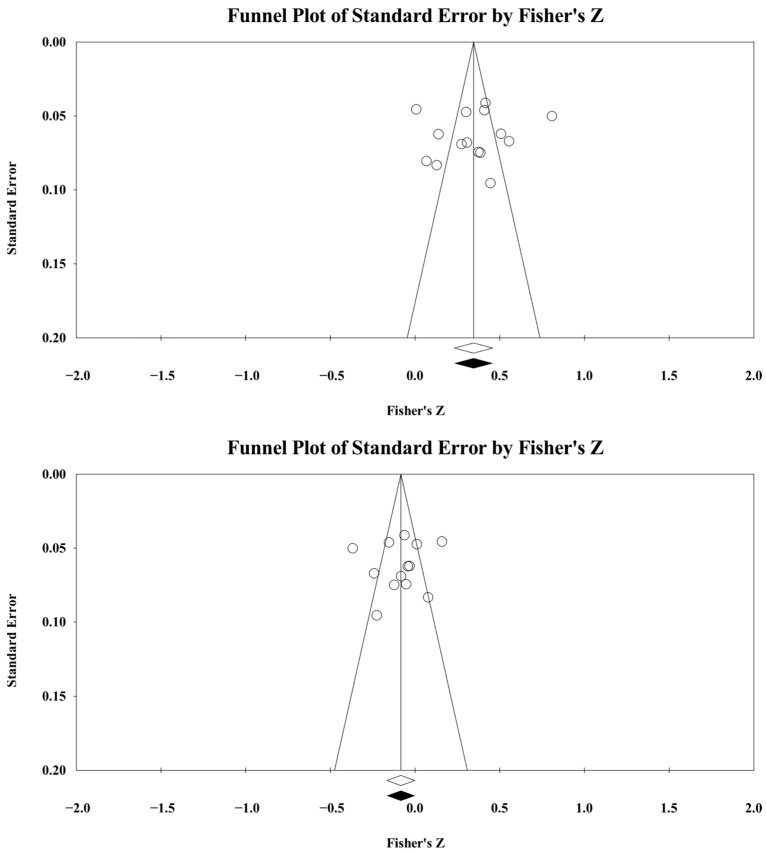
Task (**top** figure) and ego (**bottom** figure) climate and sport confidence random-effects trim and fill plot. The open circles are the data points. The clear rhombus is the mean effect size. The black rhombus is the trimmed and filled mean effect size.

**Figure 6:**
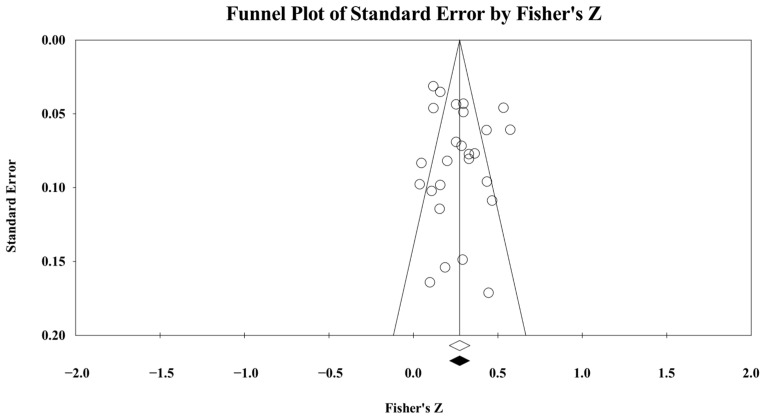

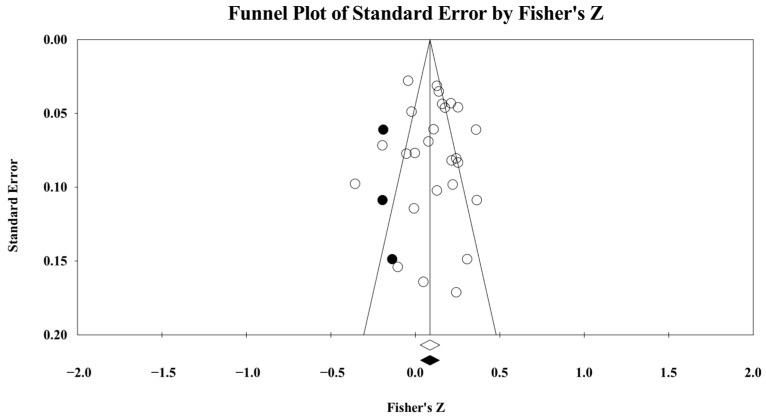
Task (**top** figure) and ego (**bottom** figure) goal orientations and sport confidence random-effects trim and fill plot. The open circles are the data points. The black circles are the trimmed and filled data points. The clear rhombus is the mean effect size. The black rhombus is the trimmed and filled mean effect size.

Table 2 presents all the summary data for the relationships examined. The individual study data, along with corresponding forest plots, are located in Figure 3, Figure 4, Figure 5 and Figure 6. The task motivational climate random effect size was positive and medium in meaningfulness. The relationships for task and ego orientations were small in meaningfulness, with the ego motivational climate relationship also small but negative in direction. The 95% confidence intervals for the task motivational climate and individual goal orientations ranged from small to medium in meaningfulness, while the 95% confidence intervals for the ego motivational climate and individual goal orientations ranged from minimal to small in meaningfulness. Notably, all AGT and sport confidence true prediction intervals crossed zero, with the task orientation prediction interval being the most promising, ranging from no relationship (−0.01) to a large positive (0.51) relationship. Heterogeneity was present, although the bias statistics indicated that, except for the relationship between task orientation and sport confidence, the relationships were either free or mostly free from bias.

### 3.4. Moderator Analyses

We set the minimum sample per moderator to at least five, as moderator analyses have historically been underpowered. Table S6 contains the results of all mixed-effects moderator analyses for the state, compared to trait, sport confidence measures, the achievement goal orientation measure (TEOSQ or POSQ), sport type, athlete level categories, and study quality. The following statistically significant (*p* < 0.05) moderator analyses were the results: task climate for state compared (*r* = 0.24) to trait (*r* = 0.41), task orientation TEOSQ (*r* = 0.31) compared to POSQ (*r* = 0.18) measurement, and study quality, lowest (*r* = 0.35), medium (*r* = 0.18), and highest (*r* = 0.24). The task climate and task orientation findings were followed up on to determine the potential overlap with the quality results. It is important to note here that all of the studies were of sufficient quality based on Kmet and colleagues’ (2004) system. Only two motivational climate studies were in the lower quality studies. However, of the 10 lower quality studies, 9 were with the TEOSQ (*r* = 0.37) and one with the POSQ (*r* = 0.16). The meta-regression analyses examining the potential impact of the sample sex makeup (i.e., % female) resulted in no significant findings (see Table S7).

### 3.5. Additional Sensitivity Analyses

The remove-one study forest plot provides a visual representation of the consistency of the results. Based on the individual point estimates, all examined relationships were very consistent, indicating that no single study had a significant impact on the overall results. The figures are found in the Supplementary Materials (see Figures S1 and S2).

## 4. Discussion

Researchers have investigated AGT and sport confidence over the last five decades. This systematic review and meta-analysis of the available published literature quantified the relationships between situational and dispositional achievement goals and sport trait and state confidence in competing athletes. In addition to the overall relationships, several moderators concerning task or mastery constructs were explored.

### 4.1. Summary of Results

When analyzing the relationship between sport confidence and achievement goal involvement, the results indicated that a task motivational climate, which is a situational measure of achievement goal involvement, had a positive medium effect size and demonstrated the greatest meaningfulness of all analyzed AGT constructs. In contrast, an ego motivational climate was negatively related to sport confidence, although the effect size was small. Task orientation and ego orientation both demonstrated small, positive effect sizes. These results suggest that operating within a task motivational climate is beneficial for building an athlete’s confidence, whereas ego motivational climates do not increase confidence and may even harm confidence levels.

It is important to note that, although the confidence intervals for the main effects did not cross zero, all prediction intervals did cross zero and were consistent with the high heterogeneity observed in the main results. Of the primary findings, the relationship between task orientation and sport confidence was the most reliable, ranging from near-zero to a large effect size. In summary, the overall findings of this meta-analysis indicated that relationships between AGT constructs and sport confidence were small to, at best, medium in magnitude, characterized by wide prediction intervals and high heterogeneity, yet with surprisingly little evidence of bias.

The moderator analyses may provide a clearer understanding of the main results. For example, although a significant difference emerged between the relationships of task climate with state and trait sport confidence, suggesting that perceptions of a task climate were more strongly related to trait sport confidence, both prediction intervals crossed zero, indicating that neither relationship is stable. Additional moderation analyses revealed that the dispositional achievement goal measure used, TEOSQ or POSQ, influenced the observed relationships. Although this finding emerged while examining study quality, a more important conclusion is that differences between the TEOSQ and POSQ relationships with sport confidence carry greater implications for future research than study quality per se, as all study quality scores were acceptable.

Furthermore, our interpretation of study sampling methods and data reporting is based on the collective characteristics of the included studies and is open to criticism or alternative interpretations. Nevertheless, the results of this quantitative review provide guidance for researchers continuing to study AGT and sport confidence, particularly with respect to the measurement of task orientation.

### 4.2. Limitations and Future Directions

Despite following the PRISMA statement throughout the research process, several limitations exist in this systematic review. The first limitation concerns the inclusion of only English-language, peer-reviewed studies within the selected databases, a limitation common to many meta-analyses in the sport psychology literature. As a result, relevant data from studies published in journals indexed entirely in non-English languages may have been missed. In addition, theses, dissertations, and conference presentations, often referred to as “grey” literature, were excluded from the outset.

Addressing these search limitations would likely have resulted in the inclusion of more studies, as sport psychology research is global and extensive (Lochbaum & Lane, 2025). Although tools such as Google Translate can be helpful, conducting systematic searches in other languages would require research teams with multilingual expertise (e.g., Lochbaum et al., 2023). Beyond search-related limitations, applicable data may also have been missed due to variations in terminology or because sport confidence data were embedded within studies primarily focused on anxiety (e.g., CSAI-2 research). For example, terms such as self-competence have often been used in this literature, either as a synonym for confidence or as a distinct construct.

Based on the results of this meta-analysis, a logical next step for advancing this line of research is to examine more closely the differences between the TEOSQ and POSQ in the task orientation–sport confidence relationship. Given that the TEOSQ task orientation–sport confidence relationship was nearly twice the magnitude of the corresponding POSQ relationship, researchers with a history of using the TEOSQ may be more encouraged to study sport confidence, whereas those using the POSQ may be less inclined to do so. As many other sport psychology measures have undergone revision, it may be time to consider the development of a TEOSQ-2 or POSQ-2. Lochbaum et al. (2016a, 2016b) previously highlighted meaningful differences between the TEOSQ and POSQ, further supporting this direction.

### 4.3. Conclusions

With five decades of research synthesized, the present findings suggest that, when athletes possess a dispositional task orientation or perceive themselves to be within a task motivational climate, a positive relationship with sport confidence is possible. However, with the substantial between-study and contextual variability presented in the results, it is also plausible that, in some settings or with differing dispositional measures, the relationship is null or even different from what is expected. Given the absence of moderated differences for the small positive relationship between ego orientation and sport confidence, as well as the negative relationship between ego climate and sport confidence, these relationships appear relatively stable. Overall, as with other AGT meta-analytic findings, our results should encourage coaches, parents, and others involved in athlete development to foster task-oriented climates and task orientations, while acknowledging that meaningful differences in perceived sport confidence may not always emerge as intended.

## Figures and Tables

**Table 1:** Study characteristics.

Study	Country	Sample Size	%F	Level	Sport	AGT	Sport Confidence
Asghar et al. (2013) Study 1	CN, DE	522	0	I	Soccer	GO	S
Asghar et al. (2013) Study 2	DE, PK	271	0	I	Field Hockey	GO	S
Assar et al. (2022)	US	418	50.5	A	Mix	GO	T
Blank et al. (2016)	AT	1265	31.3	A	Mix	GO	T
Curran et al. (2015)	UK	260	57.7	Y	Soccer	MC	T
Draugelis et al. (2014)	US	182	86.3	A	Dance	MC	T
Fernandes et al. (2012)	BR	169	17.2	M	Mix	GO	S
Fry et al. (2021)	US	467	62.3	A	Mix	MC	T
Gillham et al. (2013) Study 3	US	396	57.3	A	Mix	MC	T
Gómez-López et al. (2020)	ES	479	48.0	A	Handball	MC	S
Habeeb et al. (2023)	US	150	43.3	I	Mix	MC	T
Hall and Kerr (1997)	UK	111	32.4	Y	Fencing	GO	S
Hall et al. (1998)	UK	119	62.2	I	Cross-Country	GO	S
Kiss and Nagy (2024)	HU	293	0	M	Ice-hockey	MC	S, T
Kuan and Roy (2007)	MY	40	48.0	A	Wushu	GO	T
Martins et al. (2017)	PT	472	23.7	M	Mix	GO	T
Morales-Sanchez et al. (2022)	ES	113	44.0	I	Soccer	MC	S, T
Newton and Duda (1995)	US	107	NR	R	Tennis	GO	S
Ntoumanis and Biddle (1998)	UK	146	42.5	A	Mix Team	GO, MC	S
Ozer and Kocaeksı (2013)	TR	41	0	Y	Soccer	GO	S
Pettersen et al. (2023)	NO	156	100	E	Soccer	MC	T
Pineda-Espejel et al. (2015)	MX	211	54.7	A	Mix Team	MC, GO	S
Pineda-Espejel et al. (2016)	Mix	37	48.0	E	Gymnastics	GO	S
Pineda-Espejel et al. (2018)	Mix	171	52.6	E	Mix	GO	S
Pineda-Espejel et al. (2021)	Mix	217	48.4	E	Mix	MC	S
Reigal-Garrido et al. (2018)	ES	112	44.64	E	Beach Handball	MC	T
Rodrigues et al. (2009)	Mix	45	11.1	M	Mountaineering	GO	S
Sancho and Ruiz-Juan (2014)	ES	401	17.7	A	Track	GO	S
Santos-Rosa et al. (2022)	ES	258	100	I	Gymnastics	MC	S
Sarı and Bizan (2022)	TR	180	54	I	Mix	MC	T
Tastan et al. (2020)	TR	269	NR	A	Mix	GO	T
Vealey and Campbell (1988)	US	106	89.6	I	Figure Skating	GO	T
Voight et al. (2000)	US	196	100	I	Volleyball	GO	T
Vosloo et al. (2009)	US	151	61.6	I	Swimming	MC, GO	S
Wu et al. (2025)	CH	87	44.8	A	Track	GO	S
Zarauz-Sancho et al. (2016)	ES, MX	1795	15.3	M	Running	GO	S
Zmora et al. (2026)	IL	48	20.8	M	Basketball	GO	T

Abbreviations: AT = Austria, BR = Brazil, CN = China, DE = Germany, ES = Spain, HU = Hungary, IL = Israel, MX = Mexico, MY = Malaysia, NO = Norway, PK = Pakistan, PT = Portugal, TR = Turkey, UK = United Kingdom, US = United States of America, Y = youth, I = intermediate, A = advanced, E = elite, M = mix of levels, NR = not reported, MC = motivational climate, GO = goal orientation, S = state confidence measure, T = trait confidence measure.

**Table 2:** AGT relationships with sport confidence.

	Effect Size Statistics				Heterogeneity			Bias			
	k	ES	95% CI	95% PI	Q	*τ* ^2^	*I* ^2^	FS	Orwin	Trim/Fill	ES [95% CI]
Task climate	15	0.33	0.23, 0.43	−0.13, 0.68	192.67	0.05	92.73	2728	39	0	No change
Ego climate	13	−0.08	−0.16, −0.00	−0.38, 0.23	79.12	0.02	84.83	102	0	0	No change
Task orientation	26	0.27	0.21, 0.32	−0.01, 0.51	137.26	0.02	81.78	3785	42	0	No change
Ego orientation	26	0.11	0.06, 0.17	−0.15, 0.36	138.93	0.02	82.01	721	2	3L	0.09 [0.03, 0.14]

Abbreviations: k = number of samples, ES = effect size, CI = confidence interval, PI = prediction interval, Q = test of null hypothesis that all studies share a common effect size, τ^2^ = tau-squared, *I*^2^ = ratio of excess dispersion to total dispersion, FS = fail-safe number.

## Data Availability

All data are contained in the manuscript.

## Supplementary Materials

The following supporting information can be downloaded at https://www.mdpi.com/article/10.3390/ejihpe16020018/s1: Table S1: PRISMA checklist. Table S2: Athlete level classification system. Table S3: Kmet et al.’s (2004) quality system questions. Table S4: Risk of bias statistics with explanation. Table S5: Assessment of sport confidence measures. Table S6: Mixed-effects AGT relationships with state and trait sport confidence. Table S7: Meta-regression results with %female with each AGT construct and measures of sport confidence. Figure S1: Task climate (top figure) and ego climate (bottom figure) and sport confidence on study removed. Figure S2: Task orientation (top figure) ego orientation (bottom figure) and sport confidence on study removed.
