# Supplementary material for: Situational and Dispositional Achievement Goals’ Relationships with Measures of State and Trait Sport Confidence: A Systematic Review and Meta-Analysis
Source: Eur J Investig Health Psychol Educ. 2026 Jan 30;16(2):18. doi: 10.3390/ejihpe16020018 (PMC12939003; doi:10.3390/ejihpe16020018)
Supplement: Supplementary file 1 [file ejihpe-16-00018-s001.zip › ejihpe-4013360-supplementary.pdf]

Supplementary file contents

- **Table S1.** PRISMA Checklist
- **Table S2.** Athlete level classification system.
- **Table S3.** Kmet et al.'s (2004) quality system questions
- **Table S4.** Risk of bias statistics with explanation.
- **Table S5.** Assessment of sport confidence measures.
- **Table S6.** Mixed effects AGT relationships with state and trait sport confidence.
- **Table S7.** Meta-regression results with %female with each AGT construct and measures of sport confidence.
- **Figure S1.** Task climate (top figure) and ego climate (bottom figure) and sport confidence on study removed.
- **Figure S2.** Task orientation (top figure) ego orientation (bottom figure) and sport confidence on study removed.

Table S1. PRISMA checklist.

Section and Topic	Item #	Checklist item	Location where item is reported
**TITLE**			
Title	1	Identify the report as a systematic review.	Title
**ABSTRACT**			
Abstract	2	See the PRISMA 2020 for Abstracts checklist.	Abstract
**INTRODUCTION**			
Rationale	3	Describe the rationale for the review in the context of existing knowledge.	Introduction
Objectives	4	Provide an explicit statement of the objective(s) or question(s) the review addresses.	Study Purposes and Hypotheses
**METHODS**			
Eligibility criteria	5	Specify the inclusion and exclusion criteria for the review and how studies were grouped for the syntheses.	Section 2.1.
Information sources	6	Specify all databases, registers, websites, organisations, reference lists and other sources searched or consulted to identify studies. Specify the date when each source was last searched or consulted.	Section 2.2.
Search strategy	7	Present the full search strategies for all databases, registers and websites, including any filters and limits used.	Section 2.2.
Selection process	8	Specify the methods used to decide whether a study met the inclusion criteria of the review, including how many reviewers screened each record and each report retrieved, whether they worked independently, and if applicable, details of automation tools used in the process.	Section 2.3.
Data collection process	9	Specify the methods used to collect data from reports, including how many reviewers collected data from each report, whether they worked independently, any processes for obtaining or confirming data from study investigators, and if applicable, details of automation tools used in the process.	Section 2.3. and 2.4.
Data items	10a	List and define all outcomes for which data were sought. Specify whether all results that were compatible with each outcome domain in each study were sought (e.g. for all measures, time points, analyses), and if not, the methods used to decide which results to collect.	Section 2.3.
	10b	List and define all other variables for which data were sought (e.g. participant and intervention characteristics, funding sources). Describe any assumptions made about any missing or unclear information.	Section 2.3., 2.4.
Study risk of bias assessment	11	Specify the methods used to assess risk of bias in the included studies, including details of the tool(s) used, how many reviewers assessed each study and whether they worked independently, and if applicable, details of automation tools used in the process.	Section 2.5.
Effect measures	12	Specify for each outcome the effect measure(s) (e.g. risk ratio, mean difference) used in the synthesis or presentation of results.	Section 2.6.
Synthesis methods	13a	Describe the processes used to decide which studies were eligible for each synthesis (e.g. tabulating the study intervention characteristics and comparing against the planned groups for each synthesis (item #5)).	Section 2.6.
	13b	Describe any methods required to prepare the data for presentation or synthesis, such as handling of missing summary statistics, or data conversions.	Section 2.6.
	13c	Describe any methods used to tabulate or visually display results of individual studies and syntheses.	Section 2.6.
	13d	Describe any methods used to synthesize results and provide a rationale	Section 2.6.

Section and Topic	Item #	Checklist item	Location where item is reported
		for the choice(s). If meta-analysis was performed, describe the model(s), method(s) to identify the presence and extent of statistical heterogeneity, and software package(s) used.	
	13e	Describe any methods used to explore possible causes of heterogeneity among study results (e.g. subgroup analysis, meta-regression).	Section 2.6.
	13f	Describe any sensitivity analyses conducted to assess robustness of the synthesized results.	Section 2.6.
Reporting bias assessment	14	Describe any methods used to assess risk of bias due to missing results in a synthesis (arising from reporting biases).	Section 2.5.
Certainty assessment	15	Describe any methods used to assess certainty (or confidence) in the body of evidence for an outcome.	Section 2.6.
**RESULTS**			
Study selection	16a	Describe the results of the search and selection process, from the number of records identified in the search to the number of studies included in the review, ideally using a flow diagram.	Section 3.1.
	16b	Cite studies that might appear to meet the inclusion criteria, but which were excluded, and explain why they were excluded.	Not reported.
Study characteristics	17	Cite each included study and present its characteristics.	Table 1
Risk of bias in studies	18	Present assessments of risk of bias for each included study.	Figure 2
Results of individual studies	19	For all outcomes, present, for each study: (a) summary statistics for each group (where appropriate) and (b) an effect estimate and its precision (e.g. confidence/credible interval), ideally using structured tables or plots.	Section 3.3.
Results of syntheses	20a	For each synthesis, briefly summarise the characteristics and risk of bias among contributing studies.	Section 3.3.
	20b	Present results of all statistical syntheses conducted. If meta-analysis was done, present for each the summary estimate and its precision (e.g. confidence/credible interval) and measures of statistical heterogeneity. If comparing groups, describe the direction of the effect.	Section 3.3.
	20c	Present results of all investigations of possible causes of heterogeneity among study results.	Section 3.3.
	20d	Present results of all sensitivity analyses conducted to assess the robustness of the synthesized results.	Section 3.3.
Reporting biases	21	Present assessments of risk of bias due to missing results (arising from reporting biases) for each synthesis assessed.	Table 2
Certainty of evidence	22	Present assessments of certainty (or confidence) in the body of evidence for each outcome assessed.	Section 3.5.
**DISCUSSION**			
Discussion	23a	Provide a general interpretation of the results in the context of other evidence.	Section 4.1.
	23b	Discuss any limitations of the evidence included in the review.	Section 4.2.
	23c	Discuss any limitations of the review processes used.	Section 4.2.
	23d	Discuss implications of the results for practice, policy, and future research.	Section 4.2. and 4.3.
**OTHER INFORMATION**			
Registration and protocol	24a	Provide registration information for the review, including register name and registration number, or state that the review was not registered.	Abstract

Section and Topic	Item #	Checklist item	Location where item is reported
	24b	Indicate where the review protocol can be accessed, or state that a protocol was not prepared.	Abstract
	24c	Describe and explain any amendments to information provided at registration or in the protocol.	None
Support	25	Describe sources of financial or non-financial support for the review, and the role of the funders or sponsors in the review.	Funding statement
Competing interests	26	Declare any competing interests of review authors.	Conflict of interest statment
Availability of data, code and other materials	27	Report which of the following are publicly available and where they can be found: template data collection forms; data extracted from included studies; data used for all analyses; analytic code; any other materials used in the review.	Data availability statement

Table S2. Athlete level classification system.

Classification	Explanation
Elite	International competitions at the highest level (e.g., Olympics), professional leagues (e.g., Premier League); described by authors as elite; samples >18 years of age.
Advanced	Advanced: College athletes in all countries, youth/adolescents in country-level or professional team talent programs, and national-level competition.
Intermediate	14 to 18 years of age; USA high school, club; not identified as elite or in college; in organized training and regional-level competition.
Recreational	University intramural, adults on city teams not listed above at regional level or with extensive training schedules; sample mean age <14 unless listed in a category above; below high school.
Mix	Unable to determine a category or categories.

Table S3. Kmet et al.'s (2004) quality system questions

Question #	Question
1	Is the question or objective sufficiently described?
2	Is the design evident and appropriate for answering the study question?
3	Is the method of subject selection (and comparison group selection, if applicable) or source of information/input variables (e.g., for decision analysis) described and appropriate?
4	Are the subject (and comparison group, if applicable) characteristics or input variables/information (e.g., for decision analysis) sufficiently described?
5	If random allocation to treatment group was possible, is it described? N/A: Observational analytic studies, uncontrolled experimental studies, surveys.
6	If interventional and blinding of investigators to the intervention was possible, is it reported? N/A: Observational analytic studies, surveys, descriptive case series/reports.
7	If interventional and blinding of subjects to the intervention was possible, is it reported? N/A: Observational studies, surveys, descriptive case series/reports.
8	Are the outcome and (if applicable) exposure measure(s) well-defined and robust to measurement/misclassification bias? Are the means of assessment reported?
9	Is the sample size appropriate? N/A: Most surveys (except surveys comparing responses between groups or change over time).
10	Is the analysis described and appropriate?
11	Is some estimate of variance (e.g., confidence intervals, standard errors) reported for the main results/outcomes?
12	Has confounding been controlled for? N/A: Cross-sectional surveys of a single group, descriptive studies.
13	Are the results reported in sufficient detail?
14	Do the results support the conclusions?

Table S4. Risk of bias statistics with explanation.

Statistical test	Explanation
Classic fail-safe n (Rosenthal, 1979)	The classic fail-safe n statistic represents the number of null samples required to change a significant value into a non-significant value. We specified a one-tailed test when conducting the classic fail-safe n analysis.
Orwin's (1983) fail-safe n	Orwin's fail-safe n represents the potential number of missed studies that would move the correlation past a predetermined threshold. We chose zero as our missed study value and 0.10 or −0.10 as the threshold corresponding to the minimum cutoff point for a small effect size. The greater the value for the two fail-safe statistics, the greater the confidence that the result is safe from publication bias. For Orwin's n, only the fixed-effects analysis was provided in the CMA program.
Funnel plot with Duval and Tweedie's (2000) trim and fill	Funnel plots allow for the determination of whether the included studies are dispersed comparably on either side of the overall effect. Symmetry indicates that the retrieved studies capture the essence of all studies. The trim and fill analysis adjusts for potential missing studies. Data points filled to the right increase the effect size, while those filled to the left lower the effect size.

Table S5. Assessment of sport confidence measures.

Short Citation	Confidence Measure	Reference	State or Trait	Confidence Measure Description
Asghar et al., 2013 Study 1	Competitive State Anxiety Inventory-2	German and Chinese versions of Martens, R., Vealey, R. S., Burton, D., Bump, L., & Smith, D. E. (1990). Development and Validation of the Competitive State Anxiety Inventory-2 (CSAI-2). In R. Martens, R. S. Vealey, & D. Burton (Eds.), Competitive anxiety in sport (pp. 117–178). Human Kinetics.	S	Measures state confidence
Asghar et al., 2013 Study 2	Competitive State Anxiety Inventory-2	German and Chinese versions of Martens, R., Vealey, R. S., Burton, D., Bump, L., & Smith, D. E. (1990). Development and Validation of the Competitive State Anxiety Inventory-2 (CSAI-2). In R. Martens, R. S. Vealey, & D. Burton (Eds.), Competitive anxiety in sport (pp. 117–178). Human Kinetics.	S	Measures state confidence
Assar et al., 2022	Trait Sport-Confidence Inventory	Vealey, R.S. (1986). Conceptualization of sport-confidence and competitive orientation: Preliminary investigation and instrument development. Journal of Sport and Exercise Psychology, 8(3), 221–246.	T	Measures trait confidence
Blank et al., 2016	FEMKES (Confidence of Success subscale)	Finkenzeller T, Bernatzky P, Amesberger G. Constructing and testing a questionnaire to assess mental skills and attitudes in sport [in German].13. Hamburg: Czwalina; 2009.	T	States in description the measure is of "more general attitudes towards sport" Example item, "During a competition, I am very sure that I will reach my goals."
Curran et al., 2015	Athlete Engagement Questionnaire	Lonsdale, C., Hodge, K., & Jackson, S.A. (2007). Athlete engagement: II. Development and initial validation of the Athlete Engagement Questionnaire. International Journal of Sport Psychology, 38, 471-492.	T	Reference is "how often felt this way in the past 3 months"
Draugelis et al., 2014	Athlete Engagement Questionnaire	Lonsdale, C., Hodge, K., & Jackson, S.A. (2007). Athlete engagement: II. Development and initial validation of the Athlete Engagement Questionnaire. International Journal of Sport Psychology, 38, 471-492.	T	Reference is "how often felt this way in the past 3 months"
Fernandes et al., 2012	Revised Competitive State Anxiety Inventory-2	Cox, R. H., Martens, M., & Russell, W. D. (2003). Measuring anxiety in athletics: The revised Competitive State Anxiety Inventory-2. Journal of Sport & Exercise Psychology, 25, 519-533.	S	Measures state confidence
Fry et al., 2021	Athletic Coping Skills Inventory 28	Smith, R.E., Schutz, R.W., Smoll, F.L., & Ptacek, J. (1995). Development and	T	The confidence/achievement

		validation of a multidimensional measure of sport-specific psychological skills: The Athletic Coping Skills Inventory-28. Journal of Sport and Exercise Psychology, 17(4), 379–398. doi:10.1123/jsep.17.4.379		motivation subscale assesses athletes' perceptions of their confidence and optimal level of motivation.
Gillham et al., 2013 Study 3	Self-Confidence Inventory	Vealey, R., The Sport Confidence Inventory, Annual Meeting of the Association for the Advancement of Applied Sport Psychology, Tuscon, AZ, 2002.	T	Measure of self-confidence in sport. "How certain are you that...".
Gomez-Lopez et al., 2020	Revised Competitive State Anxiety Inventory-2	Spanish version of Cox, R. H., Martens, M., & Russell, W. D. (2003). Measuring anxiety in athletics: The revised Competitive State Anxiety Inventory-2. Journal of Sport & Exercise Psychology, 25, 519-533.	S	Measures state confidence
Habeeb et al., 2023	Athlete Engagement Questionnaire	Lonsdale, C., Hodge, K., & Jackson, S.A. (2007). Athlete engagement: II. Development and initial validation of the Athlete Engagement Questionnaire. International Journal of Sport Psychology, 38, 471-492.	T	Reference is "how often felt this way in the past 3 months"
Hall and Kerr, 1997	Competitive State Anxiety Inventory-2	Martens, R., Burton, D., Vealey, R. S., Bump, L., & Smith, D. E. (1983). Competitive State Anxiety Inventory—2 [dataset]. In PsycTESTS Dataset. American Psychological Association (APA).	S	Measures state confidence
Hall et al., 1998	Competitive State Anxiety Inventory-2	Martens, R., Burton, D., Vealey, R. S., Bump, L., & Smith, D. E. (1983). Competitive State Anxiety Inventory—2 [dataset]. In PsycTESTS Dataset. American Psychological Association (APA).	S	Measures state confidence
Kiss and Nagy, 2024	Competitive State Anxiety Inventory-2; Athletic Coping Skills Inventory 28	Martens, R., Vealey, R. S., Burton, D., Bump, L., & Smith, D. E. (1990). Development and Validation of the Competitive State Anxiety Inventory-2 (CSAI-2). In R. Martens, R. S. Vealey, & D. Burton (Eds.), Competitive anxiety in sport (pp. 117–178). Human Kinetics.; Smith, R.E., Schutz, R.W., Smoll, F.L., & Ptacek, J. (1995). Development and validation of a multidimensional measure of sport-specific psychological skills: The Athletic Coping Skills Inventory-28. Journal of Sport and Exercise Psychology, 17(4), 379–398. doi:10.1123/jsep.17.4.379	S;T	Measures state confidence; Measures trait confidence
Kuan and Roy, 2007	Psychological Performance Inventory	Loehr, J. E. (1986). Mental Toughness Training for Sports: Achieving Athletic Excellence. Lexington: Stephen Greene Press.	T	Measures trait confidence
Martins et al., 2017	Personal and Social	Adapted from Li W, Wright PM, Rukavina PB, Pickering M. Measuring Students' Perceptions of Personal and Social	T	'I believe I am capable of accomplishing my goals in sport'

	Responsibility Scale	Responsibility and the Relationship to Intrinsic Motivation in Urban Physical Education. J Teach Physl Educ, 2008; 27: 167-178 and Martins P, Rosado A, Ferreira V, Biscaia R. Examining the Validity of the Personal-social Responsibility Questionnaire among Athletes. Motriz: J Phy. E., 2015; 23: 321-328		
Morales-Sanchez, et al., 2022	Competitive State Anxiety Inventory-2; Self-confidence in Sport Competition Questionnaire	Martens, R.; Vealey, R.S.; Burton, D. Competitive Anxiety in Sport; Human Kinetics: Champaign, IL, USA, 1990.; Martínez-Romero, M.; Molina, V.M.; Oriol-Granado, X. Desarrollo y validación del cuestionario de autoconfianza en competición CACDparadeportes individuales y colectivos. Cuad. Psicol. Deporte 2016, 16, 13–20.	S;T	Measures state confidence; Measures trait confidence
Newton and Duda, 1995	Competitive State Anxiety Inventory-2	Martens, R., Burton, D., Vealey, R. S., Bump, L., & Smith, D. E. (1983). Competitive State Anxiety Inventory—2 [dataset]. In PsycTESTS Dataset. American Psychological Association (APA).	S	Measures state confidence
Ntoumanis & Biddle, 1998	Competitive State Anxiety Inventory-2	Martens, R., Burton, D., Vealey, R. S., Bump, L., & Smith, D. E. (1983). Competitive State Anxiety Inventory—2 [dataset]. In PsycTESTS Dataset. American Psychological Association (APA).	S	Measures state confidence
Ozer and Kocaeksi, 2013	Competitive State Anxiety Inventory-Children Form-2	Koruç, Z, Yılmaz, V, Turkish Adaptation of a Childeren's from of the Sport Competition Anxiety test: Scat-C, The 10th. ICHPER. SD Europian Congress, 2004, Antalya, Turkey.	S	Measures state confidence
Pettersen et al., 2023	Sport Mental Toughness Questionnaire	Sheard, M., Golby, J., and Van Wersch, A. (2009). Progress toward construct validation of the sports mental toughness questionnaire (SMTQ). Eur. J. Psychol. Assess. 25, 186–193. doi: 10.1027/1015-5759.25.3.186	T	Measure trait mental toughness, confidence a subscale
Pineda-Espejel et al., 2015	Revised Competitive State Anxiety Inventory-2	Mexican version of Cox, R. H., Martens, M. P. y Russell W. D. (2003). Measuring anxiety in athletics: The revised Competitive State Anxiety Inventory-2. Journal of Sport and Exercise Psychology, 25, 519-533.	S	Measures state confidence
Pineda-Espejel et al., 2016	Revised Competitive State Anxiety Inventory-3	Mexican version of Cox, R. H., Martens, M. P. y Russell W. D. (2003). Measuring anxiety in athletics: The revised Competitive State Anxiety Inventory-2. Journal of Sport and Exercise Psychology, 25, 519-533.	S	Measures state confidence
Pineda-Espejel et al., 2018	Revised Competitive State	Mexican version of Cox, R. H., Martens, M. P. y Russell W. D. (2003). Measuring anxiety in athletics: The revised Competitive State	S	Measures state confidence

	Anxiety Inventory-2	Anxiety Inventory-2. Journal of Sport and Exercise Psychology, 25, 519-533.		
Pineda-Espejel et al., 2021	Revised Competitive State Anxiety Inventory-2	Mexican version of Cox, R. H., Martens, M. P. y Russell W. D. (2003). Measuring anxiety in athletics: The revised Competitive State Anxiety Inventory-2. Journal of Sport and Exercise Psychology, 25, 519-533.	S	Measures state confidence
Reigal-Garrido et al., 2018	Psychological Inventory of Sports Performance	Spanish version of Loehr, J. E. (1986). Mental Toughness Training for Sports: Achieving Athletic Excellence. Lexington: Stephen Greene Press.	T	Me veo más como un perdedor que como un ganador durante las competiciones
Rodrigues et al., 2009	Competitive State Anxiety Inventory-2	Martens, R., Vealey, R. S., Burton, D., Bump, L., & Smith, D. E. (1990). Development and Validation of the Competitive State Anxiety Inventory-2 (CSAI-2). In R. Martens, R. S. Vealey, & D. Burton (Eds.), Competitive anxiety in sport (pp. 117–178). Human Kinetics.	S	Measures state confidence
Sancho and Ruiz-Juan, 2014	Revised Competitive State Anxiety Inventory-2	Cox, R. H., Martens, M., & Russell, W. D. (2003). Measuring anxiety in athletics: The revised Competitive State Anxiety Inventory-2. Journal of Sport & Exercise Psychology, 25, 519-533.	S	Measures state confidence
Santos-Rosa et al., 2022	Revised Competitive State Anxiety Inventory-2	Cox, R. H., Martens, M., & Russell, W. D. (2003). Measuring anxiety in athletics: The revised Competitive State Anxiety Inventory-2. Journal of Sport & Exercise Psychology, 25, 519-533.	S	Measures state confidence
Sari and Bizan, 2022	Athlete Engagement Questionnaire	Lonsdale, C., Hodge, K., & Jackson, S.A. (2007). Athlete engagement: II. Development and initial validation of the Athlete Engagement Questionnaire. International Journal of Sport Psychology, 38, 471-492.	T	Reference is "how often felt this way in the past 3 months"
Tastan et al., 2020	Sports Mental Toughness Questionnaire-14	Sheard, M., Golby, J., & Wersch, A. V. (2009): Progress towards construct validation of the Sports Mental Toughness Questionnaire (SMTQ). Eur. J. Psychol. Assess., 25:186-193.	T	Measures trait mental toughness with a confidence subscale
Vealey and Campbell, 1988	Trait Sport-Confidence Inventory	Vealey, R.S. (1986). Conceptualization of sport-confidence and competitive orientation: Preliminary investigation and instrument development. Journal of Sport and Exercise Psychology, 8(3), 221–246.	T	"used to measure the disposition of self-confidence" ... "used to measure precompetitive state self-confidence" ...
Voight et al., 2000	Trait Sport-Confidence Inventory	Vealey, R.S. (1986). Conceptualization of sport-confidence and competitive orientation: Preliminary investigation and instrument development. Journal of Sport and Exercise Psychology, 8(3), 221–246.	T	Measures trait confidence

Vosloo et al., 2009	Competitive State Anxiety Inventory-2	Martens, R., Vealey, R. S., Burton, D., Bump, L., & Smith, D. E. (1990). Development and Validation of the Competitive State Anxiety Inventory-2 (CSAI-2). In R. Martens, R. S. Vealey, & D. Burton (Eds.), Competitive anxiety in sport (pp. 117–178). Human Kinetics.	S	Measures state confidence
Wu et al., 2025	Adapted CSAI-2	Martens, R., Vealey, R. S., Burton, D., Bump, L., & Smith, D. E. (1990). Development and Validation of the Competitive State Anxiety Inventory-2 (CSAI-2). In R. Martens, R. S. Vealey, & D. Burton (Eds.), Competitive anxiety in sport (pp. 117–178). Human Kinetics.<br><br>Wish, B. (1994). Revision of the Chinese norm of the competitive state anxiety Inventory-2 (CSAI-2). *Psychol. Sci. 6,* 358–362.	S	Measures state confidence
Zarauz-Sancho et al., 2016	Revised Inventory of Competitive Anxiety-2	Spanish version of Cox, R. H., Martens, M. P. y Russell W. D. (2003). Measuring anxiety in athletics: The revised Competitive State Anxiety Inventory-2. Journal of Sport and Exercise Psychology, 25, 519-533.	S	Measures state confidence
Zmora et al., 2026	Trait Sport-Confidence Inventory	Vealey, R.S. (1986). Conceptualization of sport-confidence and competitive orientation: Preliminary investigation and instrument development. Journal of Sport and Exercise Psychology, 8(3), 221–246.<br><br>Zmora, G., Trowbridge, C., Brener, S., & Been, E. (2024). Israeli adaptation and development of the Trait Sport-Confidence Inventory (TSCI-IL). *The Spirit of Sport, 10,* 71–81.	T	Measures trait confidence

Table S6. Mixed effects AGT relationships with state and trait sport confidence.

AGT Construct	Moderator	*k*	ES	95% CI	95% PI	Q, *p*-value
Task climate	State sport confidence	7	0.24	0.11, 0.36	-0.20, 0.60	
	Trait sport confidence	9	0.41	0.29, 0.52	-0.07, 0.73	3.94, 0.047
Ego climate	State sport confidence	6	-0.03	-0.14, 0.09	-0.38, 0.34	
	Trait sport confidence	8	-0.14	-0.24, -0.04	-0.45, 0.19	2.27, 0.131
Task orientation	State sport confidence	19	0.25	0.17, 0.31	-0.00, 0.47	
	Trait sport confidence	7	0.32	0.21, 0.41	-0.02, 0.60	1.34, 0.247
Ego orientation	State sport confidence	18	0.12	0.06, 0.17	-0.08, 0.30	
	Trait sport confidence	8	0.11	-0.03, 0.24	-0.35, 0.53	0.01, 0.918
Task orientation	TEOSQ	17	0.31	0.24, 0.38	0.03, 0.55	
	POSQ	7	0.18	0.12, 0.24	-0.01, 0.36	7.61, 0.006
Ego orientation	TEOSQ	16	0.09	-0.00, 0.18	-0.28, 0.43	
	POSQ	7	0.17	0.12, 0.19	0.11, 0.20	1.67, 0.196
Task orientation	Individual sports	10	0.21	0.13, 0.29	-0.02, 0.42	
	Team sports	11	0.25	0.16, 0.33	-0.06, 0.51	0.41, 0.523
Ego orientation	Individual sports	9	0.11	-0.00, 0.23	-0.25, 0.45	
	Team sports	11	0.13	0.06, 0.19	-0.09, 0.33	0.03, 0.858
Task climate	Advanced, elite	9	0.31	0.13, 0.46	-0.36, 0.77	
	Intermediate, rec, youth	5	0.36	0.23, 0.48	-0.16, 0.72	0.26, 0.610
Ego climate	Advanced, elite	7	-0.09	-0.24, 0.06	-0.56, 0.42	
	Intermediate, rec, youth	5	-0.08	-0.16, 0.01	-0.35, 0.20	0.02, 0.884
Task orientation	Advanced, elite	9	0.28	0.19, 0.36	-0.01, 0.52	
	Intermediate, rec, youth	12	0.21	0.15, 0.27	0.02, 0.38	1.52, 0.218
Ego orientation	Advanced, elite	10	0.13	0.03, 0.23	-0.20, 0.44	
	Intermediate, rec, youth	11	0.10	0.01, 0.18	-0.20, 0.38	0.28, 0.595
Task orientation	Lowest quality	10	0.35	0.26, 0.44	0.01, 0.62	
	Medium quality	7	0.18	0.12, 0.24	0.01, 0.34	
	Highest quality	9	0.24	0.16, 0.31	0.00, 0.45	8.86, 0.012
Ego orientation	Lowest quality	10	0.11	-0.01, 0.22	-0.29, 0.48	
	Medium quality	7	0.12	0.04, 0.19	-0.11, 0.33	
	Highest quality	9	0.11	0.01, 0.21	-0.22, 0.42	0.03, 0.985

Abbreviations: TEOSQ = Task and Ego Orientation in Sport Questionnaire, POSQ = Perception of Success Questionnaire, k = number of samples, ES = effect size, CI = confidence interval, PI = prediction interval, Q = total between statistic.

Table S7. Meta-regression results with %female with each AGT construct and measures of sport confidence.

AGT Construct	Covariate	Coefficient	*SE*	95% LL	95% UL	*Z*	*p*-value 1-sided	R^2 analog
Task climate	Intercept	0.47	0.15	0.17	0.76	3.13	< 0.001	
	%Female	-0.00	0.00	-0.01	0.00	-0.92	0.17	0.00<br>Computed value -0.08
Ego climate	Intercept	-0.07	0.11	-0.29	0.14	-0.64	0.26	
	%Female	0.00	0.00	-0.00	0.00	0.11	0.45	0.00<br>Computed value -0.15
Task orientation	Intercept	0.30	0.05	0.20	0.40	5.92	< 0.001	
	%Female	-0.00	0.00	-0.00	0.00	-0.74	0.22	0.00<br>Computed value -0.08
Ego orientation	Intercept	0.12	0.03	0.06	0.19	3.48	< 0.001	
	%Female	-0.00	0.00	-0.00	0.00	0.01	0.49	0.00<br>Computed value -0.08

Abbreviations: AGT = Achievement Goal Theory, SE = standard error, LL = lower limit, UL = uppler limit.

Study name	Subgroup within study	Point	Lower limit	Upper limit	Z-Value	p-Value
		Statistics with study removed				
Curran et al. 2015	Task Climate	0.322	0.211	0.424	5.482	0.000
Draugelis et al. 2014	Task Climate	0.330	0.220	0.433	5.606	0.000
Fry et al. 2021	Task Climate	0.328	0.213	0.434	5.384	0.000
Gillham et al. 2013 Study 3	Task Climate	0.301	0.217	0.381	6.715	0.000
Gomez-Lopez et al. 2020	Task Climate	0.355	0.263	0.441	7.131	0.000
Habeeb et al. 2023	Combined	0.335	0.221	0.440	5.522	0.000
Kiss & Nagy 2024	Combined	0.327	0.211	0.434	5.321	0.000
Morales-Sanchez et al. 2022	Combined	0.319	0.209	0.420	5.499	0.000
Ntoumanis & Biddle 1998	Task Climate	0.345	0.238	0.444	6.027	0.000
Pettersen et al. 2023	Task Climate	0.349	0.244	0.446	6.180	0.000
Pineda-Espejel et al. 2015	Task Climate	0.337	0.226	0.438	5.717	0.000
Pineda-Espejel et al. 2021	Task Climate	0.335	0.224	0.437	5.662	0.000
Reigal et al. 2018	Task Climate	0.326	0.217	0.428	5.607	0.000
Santos-Rosa et al. 2022	Task Climate	0.345	0.238	0.444	6.018	0.000
Sari & Bizan 2022	Task Climate	0.330	0.219	0.432	5.597	0.000

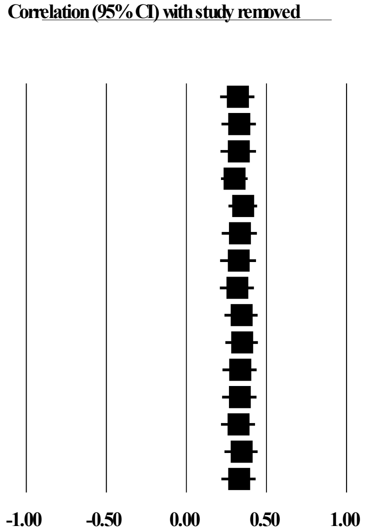

Study name	Subgroup within study	Point	Lower limit	Upper limit	Z-Value	p-Value
		Statistics with study removed				
Curran et al. 2015	Ego Climate	-0.088	-0.175	0.001	-1.941	0.052
Draugelis et al. 2014	Ego Climate	-0.086	-0.173	0.002	-1.918	0.055
Fry et al. 2021	Ego Climate	-0.077	-0.166	0.013	-1.686	0.092
Gillham et al. 2013 Study 3	Ego Climate	-0.056	-0.123	0.011	-1.641	0.101
Gomez-Lopez et al. 2020	Ego Climate	-0.106	-0.177	-0.034	-2.876	0.004
Habeeb et al. 2023	Combined	-0.092	-0.180	-0.003	-2.028	0.043
Kiss & Nagy 2024	Combined	-0.086	-0.177	0.007	-1.811	0.070
Morales-Sanchez et al. 2022	Combined	-0.071	-0.155	0.014	-1.627	0.104
Ntoumanis & Biddle 1998	Ego Climate	-0.095	-0.179	-0.010	-2.187	0.029
Pineda-Espejel et al. 2015	Ego Climate	-0.084	-0.171	0.005	-1.856	0.063
Reigal et al. 2018	Ego Climate	-0.074	-0.159	0.012	-1.696	0.090
Santos-Rosa et al. 2022	Ego Climate	-0.087	-0.175	0.002	-1.921	0.055
Sari & Bizan 2022	Ego Climate	-0.081	-0.167	0.007	-1.799	0.072

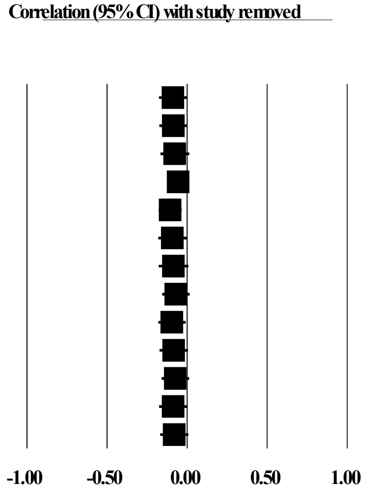

Figure S1. Task climate (top figure) and ego climate (bottom figure) and sport confidence on study removed. Figure citations: Curran et al. 2015; Draugelis et al. 2014; Fry et al. 2021; Gillham et al. 2013; Gomez-Lopez et al. 2020; Habeeb et al. 2023; Kiss and Nagy 2024; Morales-Sanchez et al. 2022; Ntoumanis and Biddle 1998; Pettersen et al. 2023; Pineda-Espejel et al. 2015; Pineda-Espejel et al. 2021; Reigal et al. 2018; Santos-Rosa et al. 2022; Sari and Bizan 2022.

Subgroup within study	Study name	Statistics with study removed				
		Point	Lower limit	Upper limit	Z-Value	p-Value
Task Orientation	Ashgar 2013 Study 1	0.268	0.206	0.327	8.277	0.000
Task Orientation	Ashgar 2013 Study 2	0.254	0.200	0.307	8.864	0.000
Task Orientation	Assar et al. 2022	0.266	0.205	0.325	8.290	0.000
Task Orientation	Fernandes et al. 2012	0.265	0.205	0.322	8.424	0.000
Task Orientation	Hall & Kerr 1997	0.261	0.203	0.318	8.441	0.000
Combined	Hall et al. 1998	0.274	0.215	0.331	8.808	0.000
Task Orientation	Kuan & Roy 2007	0.271	0.212	0.327	8.778	0.000
Task Orientation	Martins et al. 2017	0.255	0.202	0.306	9.180	0.000
Task Orientation	Newton & Duda 1995	0.275	0.217	0.331	8.954	0.000
Task Orientation	Ntoumanis & Biddle 1998	0.275	0.217	0.331	8.972	0.000
Combined	Ozer & Kocaeksi 2013	0.270	0.212	0.327	8.705	0.000
Task Orientation	Pineda-Espejel et al. 2015	0.268	0.208	0.326	8.466	0.000
Task Orientation	Pineda-Espejel et al. 2016	0.264	0.205	0.320	8.557	0.000
Task Orientation	Pineda-Espejel et al. 2018	0.263	0.204	0.321	8.409	0.000
Task Orientation	Rodrigues et al. 2009	0.269	0.210	0.326	8.688	0.000
Combined	Sancho & Ruiz-Juan 2014	0.272	0.212	0.331	8.541	0.000
Task Orientation	Tastan et al. 2020	0.260	0.201	0.317	8.421	0.000
Task Orientation	Vealey & Campbell 1988	0.271	0.212	0.328	8.690	0.000
Task Orientation	Voight et al. 2000	0.266	0.207	0.324	8.438	0.000
Task Orientation	Vosloo et al. 2009	0.270	0.210	0.327	8.593	0.000
Task Orientation	Wu et al. 2025	0.261	0.202	0.318	8.461	0.000
Task Orientation	Zarauz-Sancho et al. 2016 S1	0.275	0.217	0.331	9.003	0.000
Task Orientation	Zarauz-Sancho et al. 2016 S2	0.272	0.214	0.329	8.785	0.000
Task Orientation	Zarauz-Sancho et al. 2016 S3	0.266	0.204	0.325	8.229	0.000
Task Orientation	Zarauz-Sancho et al. 2016 S4	0.265	0.205	0.322	8.435	0.000
Task Orientation	Zmora et al. 2026	0.267	0.208	0.323	8.599	0.000

Correlation (95% CI) with study removed

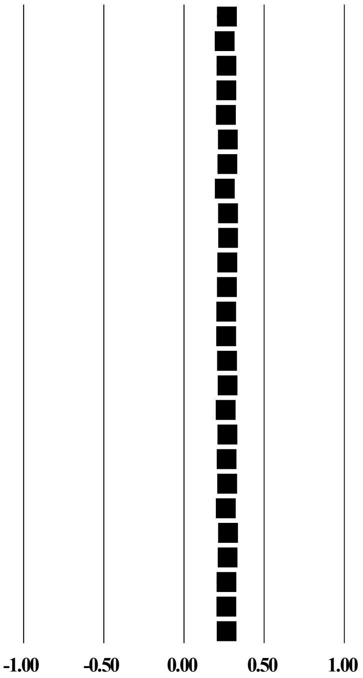

Subgroup within study	Study name	Statistics with study removed				
		Point	Lower limit	Upper limit	Z-Value	p-Value
Ego Orientation	Ashgar 2013 Study 1	0.110	0.050	0.169	3.566	0.000
Ego Orientation	Ashgar 2013 Study 2	0.112	0.053	0.171	3.688	0.000
Ego Orientation	Assar et al. 2022	0.119	0.060	0.176	3.957	0.000
Ego Orientation	Blank et al. 2016	0.121	0.066	0.175	4.263	0.000
Ego Orientation	Fernandes et al. 2012	0.119	0.061	0.176	3.992	0.000
Combined	Hall et al. 1998	0.109	0.049	0.168	3.562	0.000
Ego Orientation	Kuan & Roy 2007	0.113	0.056	0.171	3.825	0.000
Ego Orientation	Martins et al. 2017	0.105	0.047	0.163	3.538	0.000
Ego Orientation	Newton & Duda 1995	0.128	0.074	0.181	4.634	0.000
Ego Orientation	Ntoumanis & Biddle 1998	0.107	0.048	0.164	3.577	0.000
Combined	Ozer & Kocaeksi 2013	0.116	0.058	0.173	3.887	0.000
Ego Orientation	Pineda-Espejel et al. 2015	0.114	0.054	0.172	3.750	0.000
Ego Orientation	Pineda-Espejel et al. 2016	0.110	0.052	0.167	3.705	0.000
Ego Orientation	Pineda-Espejel et al. 2018	0.117	0.058	0.175	3.893	0.000
Ego Orientation	Rodrigues et al. 2009	0.117	0.059	0.174	3.960	0.000
Combined	Sancho & Ruiz-Juan 2014	0.111	0.049	0.171	3.534	0.000
Ego Orientation	Tastan et al. 2020	0.101	0.045	0.156	3.530	0.000
Ego Orientation	Vealey & Campbell 1988	0.108	0.050	0.166	3.628	0.000
Ego Orientation	Voight et al. 2000	0.125	0.070	0.180	4.408	0.000
Ego Orientation	Vosloo et al. 2009	0.108	0.050	0.166	3.607	0.000
Ego Orientation	Wu et al. 2025	0.104	0.047	0.161	3.549	0.000
Ego Orientation	Zarauz-Sancho et al. 2016 S1	0.111	0.049	0.172	3.508	0.000
Ego Orientation	Zarauz-Sancho et al. 2016 S2	0.112	0.053	0.169	3.724	0.000
Ego Orientation	Zarauz-Sancho et al. 2016 S3	0.107	0.048	0.166	3.536	0.000
Ego Orientation	Zarauz-Sancho et al. 2016 S4	0.107	0.049	0.165	3.581	0.000
Ego Orientation	Zmora et al. 2026	0.108	0.050	0.165	3.644	0.000

Correlation (95% CI) with study removed

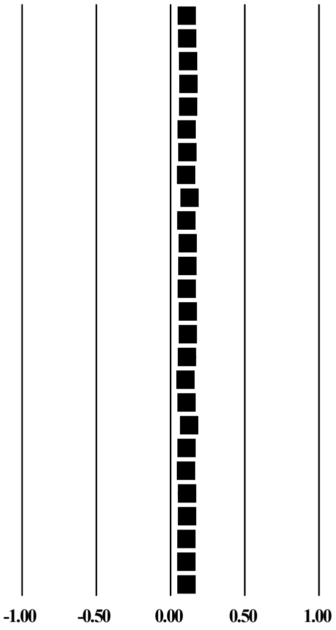

Figure S2. Task orientation (top figure) ego orientation (bottom figure) and sport confidence on study removed. Figure citations: Ashgar 2013; Assar et al. 2022; Fernandes et al. 2012; Hall and Kerr 1997; Hall et al. 1998; Kuan and Roy 2007; Martins et al. 2017; Newton and Duda 1995; Ntoumanis and Biddle 1998; Ozer and Kocaeksi 2013; Pineda-Espejel et al. 2015; Pineda-Espejel et al. 2016; Pineda-Espejel et al. 2018; Rodrigues et al. 2009; Sancho & Ruiz-Juan 2014; Tastan et al. 2020; Vealey & Campbell 1988; Voight et al. 2000; Vosloo et al. 2009; Wu et al. 2025; Zarauz-Sancho et al. 2016; Zarauz-Sancho et al. 2016; Zmora et al. 2026.
